# Vitamin A deficiency modulates iron metabolism independent of hemojuvelin (*Hfe2*) and bone morphogenetic protein 6 (*Bmp6*) transcript levels

**DOI:** 10.1186/s12263-016-0519-4

**Published:** 2016-03-17

**Authors:** Juliana Frossard Ribeiro Mendes, Egle Machado de Almeida Siqueira, João Gabriel Marques de Brito e Silva, Sandra Fernandes Arruda

**Affiliations:** 1 Postgraduate Program in Human Nutrition, Faculty of Health Sciences, University of Brasília, Campus Universitário Darcy Ribeiro, POBox 70910-900, Brasília, DF Brazil; 2 Cell Biology Department of Biological Sciences Institute, University of Brasília, Campus Universitário Darcy Ribeiro, POBox 70910-900, Brasília, DF Brazil; 3 Nutrition Department of Health Sciences Faculty, University of Brasília, Campus Universitário Darcy Ribeiro, POBox 70910-900, Brasília, DF Brazil; 4 Instituto de Ciências Biológicas, Departamento de Biologia Celular, Laboratório de Bioquímica da Nutrição, Universidade de Brasília, Campus Universitário Darcy Ribeiro, Bloco J, 1 Andar. Asa Norte, Brasília, Distrito Federal CEP: 70910-900 Brasil

**Keywords:** Vitamin A deficiency, Iron status, Hemojuvelin, Bone morphogenetic protein 6, Hepcidin

## Abstract

**Background:**

Considering that vitamin A deficiency modulates hepcidin expression and consequently affects iron metabolism, we evaluated the effect of vitamin A deficiency in the expression of genes involved in the hemojuvelin (HJV)-bone morphogenetic protein 6 (BMP6)-small mothers against decapentaplegic protein (SMAD) signaling pathway.

**Methods:**

Male Wistar rats were treated: control AIN-93G diet (CT), vitamin A-deficient diet (VAD), iron-deficient diet (FeD), vitamin A- and iron-deficient diet (VAFeD), or 12 mg all-*trans* retinoic acid (atRA)/kg diet.

**Results:**

Vitamin A deficiency (VAD) increased hepatic *Bmp6* and *Hfe2* mRNA levels and down-regulated hepatic *Hamp*, *Smad7*, *Rarα*, and intestinal *Fpn1* mRNA levels compared with the control. The FeD rats showed lower hepatic *Hamp*, *Bmp6*, and *Smad7* mRNA levels compared with those of the control, while in the VAFeD rats only *Hamp* and *Smad7* mRNA levels were lower than those of the control. The VAFeD diet up-regulated intestinal *Dmt1* mRNA levels in relation to those of the control. The replacement of retinyl ester by atRA did not restore hepatic *Hamp* mRNA levels; however, the hepatic *Hfe2*, *Bmp6*, and *Smad7* mRNA levels were similar to the control. The atRA rats showed an increase of hepatic *Rarα* mRNA levels and a reduction of intestinal *Dmt1* mRNA and *Fpn1* levels compared with those of the control.

**Conclusions:**

The HJV-BMP6-SMAD signaling pathway that normally activates the expression of hepcidin in iron deficiency is impaired by vitamin A deficiency despite increased expression of liver *Bmp6* and *Hfe2* mRNA levels and decreased expression of *Smad7* mRNA. This response may be associated to the systemic iron deficiency and spleen iron retention promoted by vitamin A deficiency.

**Electronic supplementary material:**

The online version of this article (doi:10.1186/s12263-016-0519-4) contains supplementary material, which is available to authorized users.

## Background

Iron deficiency affects approximately 20 % of the world population and is the most prevalent nutritional deficiency worldwide (Martínez-Navarrete et al. 2002; Brasil 2007; Assunção et al. 2007). The condition is often considered together with vitamin A deficiency, which affects approximately 250 million people (Strube et al 2002; Zimmermann et al. 2006).

Hepcidin, a peptide hormone secreted by the liver, is an essential molecule for systemic iron homeostasis regulation. The primary role of hepcidin is to control the amount of iron that is released into the circulation from enterocytes and spleen macrophages. Hepcidin binds to ferroportin, which is an iron exporter protein found in most body cell membranes, and triggers the internalization of ferroportin, ubiquitination, and subsequent lysosomal degradation. The result is the prevention of the iron release from enterocytes and splenic macrophages. Hepcidin gene expression is regulated by several factors, including body iron status, inflammation, erythropoiesis, and hypoxia (Evstatiev and Gasche 2012).

When body iron stores are high, hepcidin expression is increased, which consequently inhibits iron mobilization from tissues and iron absorption from the diet (Anderson et al. 2009; Hentze et al. 2010). The pathway that modulates hepcidin expression in response to intracellular iron status appears to be dependent on the hemojuvelin (HJV) protein and bone morphogenetic protein 6 (BMP6). The binding of the BMP6 to the BMP receptor (BMPR) and its essential co-receptor HJV leads to the phosphorylation of small mothers against decapentaplegic protein (SMAD) 1, 5, and 8. Phosphorylated SMAD 1/5/8 forms a complex with SMAD 4 that is translocated to the nucleus before activating the hepcidin promoter; this process induces iron retention in tissues (Hentze et al. 2010; Lee et al. 2010; Zhang et al. 2010; Zhang 2010). The BMP6 pathway also induces negative regulators of hepcidin, such as SMAD 7, so that a negative feedback loop that controls hepcidin expression is established. This mechanism prevents excessive hepcidin production, which in turn could lead to iron deficiency (Evstatiev and Gasche 2012).

Recent studies suggest that the metabolism of vitamin A and iron is interrelated (Citelli et al. 2012; Jiang et al. 2012; Tsuchiya et al. 2009). In vitamin A-deficient organisms, systemic iron availability appears to be significantly reduced and iron is accumulated in tissues, especially in the spleen (Tsuchiya et al. 2009; Arruda et al. 2009). Although preliminary data clearly show that vitamin A affects iron homeostasis (Arruda et al. 2009), the mechanism by which retinoids modulate systemic iron homeostasis is unclear. Several physiological processes, such as cell proliferation and differentiation, are modulated by retinoids. The all-trans retinoic acid (atRA), which is the most active form of retinoids, modulates the expression of several genes at the transcriptional level by binding to nuclear receptors, the retinoic acid receptor (RAR), and the retinoid X receptor (RXR), which are transcription factors (Ziouzenkova and Plutzky 2008; Theodosiou et al. 2010).

To explore how vitamin A deficiency alters iron metabolism, the present study evaluated the expression of genes involved in the HJV-BMP6-SMAD signaling pathway. The study also considered the possible involvement of retinoic acid and retinoid X nuclear receptors in the maintenance of iron homeostasis that is mediated by vitamin A.

## Methods

### Animals

Thirty 21-day-old male Wistar rats (65.7 ± 5.5 g) were housed individually in stainless steel cages and were maintained under a 12 h light cycle at 22 ± 2 °C with free access to water, and food access was allowed only during the dark cycle. The present study was approved by the Animal Care and Use Committee of the University of Brasília (UnBDoc 52606/2011). All applicable national and institutional guidelines for the care and use of animals were followed.

After 7 days of acclimatization, rats were randomly assigned to five groups (six rats per group). The control group (CT) was fed with an AIN-93G diet (Reeves et al. 1993), containing 4000 UI vitamin A/kg; the vitamin A-deficient group (VAD) was fed with an AIN-93G diet without a source of vitamin A; the vitamin A- and iron-deficient group (VAFeD) was fed with an AIN-93G diet without sources of vitamin A and iron; the iron-deficient group (FeD) was fed with an AIN-93G diet without a source of iron; and the retinoic acid group (atRA) was fed with an AIN-93G diet containing 12 mg of atRA/kg as the only source of vitamin A. The animals were weighed weekly, and food intake was recorded daily. After 59 days of feeding the experimental diets, the animals were denied food for 14 h and sacrificed after being anesthetized with 3 % isoflurane. Blood was collected by cardiac puncture into tubes containing 7.0 % ethylene-diaminetetraacetic acid (21 μL/mL of blood). The liver, spleen, and small intestine (a 1-cm length of small intestine distal to the pylorus and a 1-cm length proximal to the ileocecal valve were excised, and the lumen was rinsed with saline) were excised and washed in cold 0.9 % NaCl. The samples were frozen in liquid nitrogen (N_2_) and stored at −80 °C.

### RNA extraction and reverse transcription-polymerase chain reaction analysis (qRT-PCR)

The extraction of total RNA, purity and integrity of the RNA samples, and the cDNA synthesis were performed as described previously (Cunha et al. 2014).

Quantitative real-time PCR was performed using the Fast SYBR Green Master Mix 2x reagent (Applied Biosystems, Foster City, CA, USA) with 2.0 μL of cDNA diluted 50 times (corresponding to 2 ng of total RNA). The final volumes were 10 and 5 μL Fast SYBR Green Master Mix and 10 μmol/L (final concentration) of each primer. The following primers were used for each target genomic region: for hepcidin, *Hamp* TGATGCTGAAGCGAAGGA (forward) and TGTGTTGAGAGGTCAGGAC (reverse); for hemochromatosis type 2 (juvenile) homolog, *Hfe2* GTAGCATCGGGAGCCAAC (forward) and TCAAAGGCTGCAGGAAGATT (reverse); for bone morphogenetic protein 6, *Bmp6* GACAGCAGAGTCGCAATCG (forward) and AGCTCACGTAAAGCTCATGC (reverse); for SMAD family member 7, *Smad7* AGAGGCTGTGTTGCTGTG (forward) and CATCGGGTATCTGGAGTAAGG (reverse); for retinoic acid receptor alpha, *Rarα* ACCATTGCCGACCAGATTACCC (forward) and AAGGTCATTGTGTCTTGCTCAGGT (reverse); for retinoid X receptor beta, *Rxrβ* CTTCCCAGTCATCAGTTCTTCC (forward) and GGTGGCTTCACATCTTCAGG (reverse); for divalent metal transporter 1, *Dmt1* or solute carrier family 11 member 2, *Scl11a2* CTGATTTACAGTCTGGAGCAG (forward) and CACTTCAGCAAGGTGCAA (reverse); for ferroportin 1, *Fpn1* or solute carrier family 40 member 1, *Slc40a1* TTCCGCACTTTTCGAGATGG (forward) and TACAGTCGAAGCCCAGGACTGT (reverse); and for β-actin, *Actb* GTCGTACCACTGGCATTGTG (forward) and CTCTCAGCTGTGGTGGTGAA (reverse). Quantitative PCR was performed using a 7500 Fast Real-Time PCR System (Applied Biosystems, Cingapura) with 40 cycles at 95 °C for 20 s, 60 °C for 3 s, and 60 °C for 20 s. “The expression of all genes was normalized to the expression of the housekeeping gene β-actin, and the reactions were run in triplicate. The amplification efficiency was determined from the slope obtained from the standard curve relating log [transcribed mRNA] and variation threshold cycle (*C*_T_) with the equation *E* = (10 − 1/slope − 1) × 100, and a slope value of the regression line plot of *C*_T_ values versus log of input nucleic acid of approximately −3.32 was considered an efficient reaction. PCR efficiency was between 102 and 109 % for all primers. A standard curve was plotted for each gene, which correlated the Δ*C*_T_ (*C*_T_ target − *C*_T_ reference) versus the log of the cDNA amount, and a slope value of the regression line plot of ∆*C*_T_ values versus log of input nucleic acid of less than 0.1 was used as a general criterion to accept the validation of the experiment. Based on the data obtained previously, a 1:50 dilution of cDNA was used to analyze qRT-PCR, where the efficiency was higher than 99 %. Melting curve analysis was used to examine the specificity of the products generated for each primer set. The comparative *C*_T_ method was used to quantitate the abundance of the target gene mRNA and is given by ${2}^{-\varDelta \varDelta {C}_{\mathrm{T}}}$.” This method was performed as described in the tutorial, “Guide to Performing Relative Quantitation of Gene Expression Using Real-Time Quantitative PCR” (Part #: 4371095 Rev B, Applied Biosystems).

### Statistical analysis

Statistical analyses were performed using SPSS software (version 19.0, SPSS Inc., Chicago, IL, USA). The normality of data distribution was tested by the Kolmogorov-Smirnov test. Differences between treatment groups were tested by independent sample *t* test. In all tests, a value of *p* < 0.05 was considered statistically significant.

## Results

### The effect of vitamin A deficiency and atRA on the expression of mRNAs encoding proteins involved in iron homeostasis: *Hamp*, *Hfe2*, *Bmp6*, and *Smad7*

**Fig. 1:**
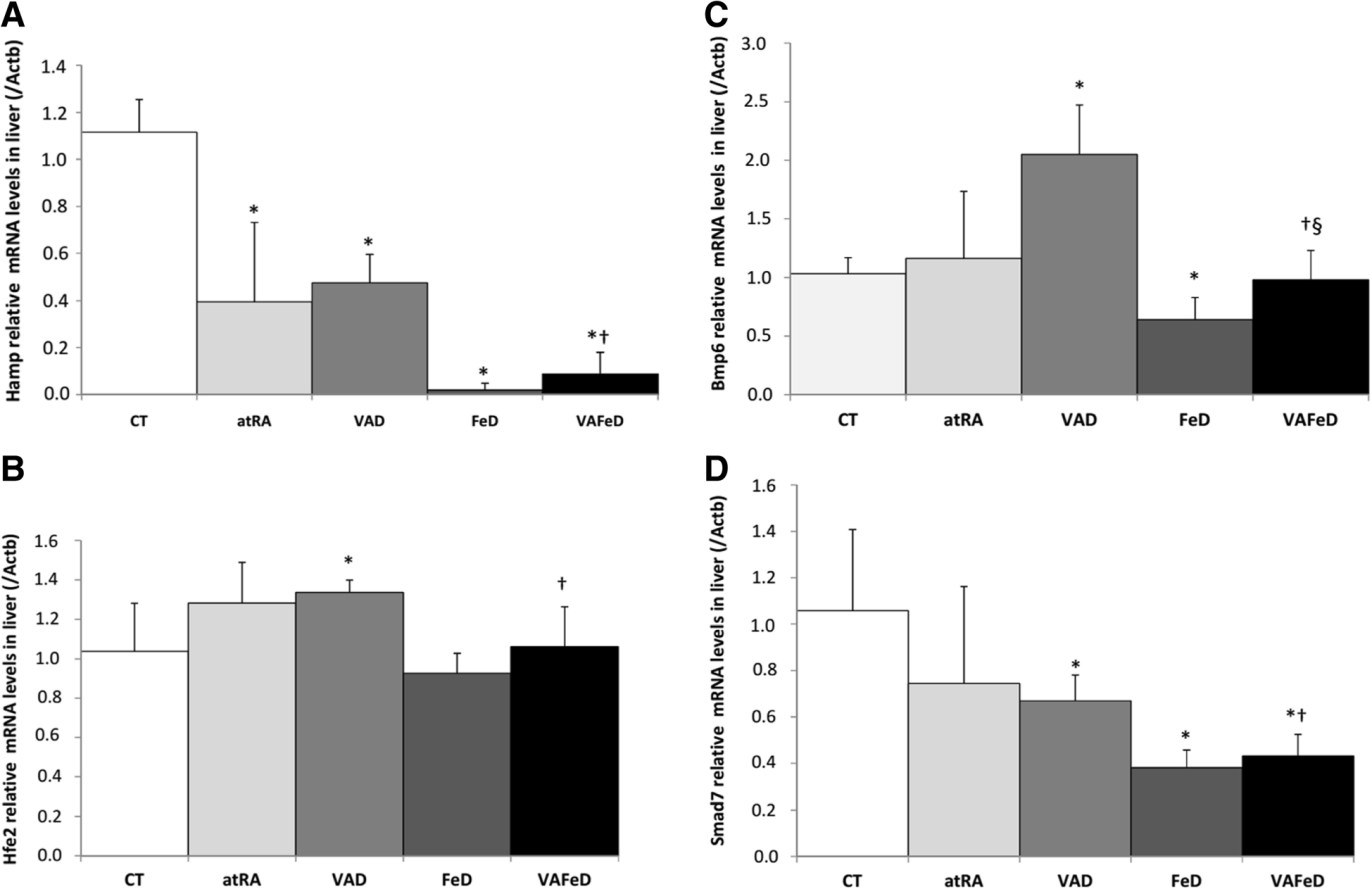
Relative transcript levels of hepcidin (*Hamp*) (**a**), hemochromatosis type 2 (juvenile) homolog (*Hfe2*) (**b**), bone morphogenetic protein 6 (*Bmp6*) (**c**), and SMAD family member 7 (*Smad7*) (**d**) in the livers normalized to β-actin (Actb) values of rats treated with diets containing different sources and amounts of vitamin A and iron for 59 days. Mean ± standard deviation, *p < 0.05 versus the CT group, †*p* < 0.05 versus the VAD group, §*p* < 0.05 versus the FeD group

Figure 1 shows the relative transcript levels of hepcidin (*Hamp*), hemochromatosis type 2 (juvenile) homolog (*Hfe2*), bone morphogenetic protein 6 (*Bmp6*), and SMAD family member 7 (*Smad7*) in rat livers, normalized to β-actin (*Actb*) values. Compared to the control group, vitamin A deficiency down-regulated *Hamp* (2.3-fold, *p* < 0.001) and *Smad7* (1.6-fold, *p* = 0.027) mRNA levels in the liver and up-regulated hepatic *Hfe2* (1.2-fold, *p* = 0.026) and *Bmp6* mRNA levels (2.0-fold, *p* = 0.0013). The consumption of atRA, as the only source of dietary vitamin A, resulted in mRNA levels of *Hfe2*, *Bmp6*, and *Smad7* be similar to those values obtained in the control group; however, the atRA diet failed to reverse the inhibitory effect of vitamin A deficiency on *Hamp* mRNA levels (2.8-fold, *p* = 0.002) in the liver. The iron-deficient diet (FeD) down-regulated hepatic levels of *Hamp* (58.7-fold), *Bmp6* (1.6-fold), and *Smad7* mRNA (2.8-fold, *p* = 0.000, 0.002, and 0.005, respectively) compared to those of the control group, and no difference was observed in relation to hepatic *Hfe2* mRNA levels. The VAFeD group showed lower hepatic levels of *Hamp* and *Smad7* mRNA (12.6-fold and 2.4-fold, *p* = 0.000 and 0.006, respectively) relative to the control group, but no difference was observed in the *Hfe2* and *Bmp6* mRNA levels, between these two groups. The VAFeD diet also significantly reduced the hepatic *Hamp*, *Hfe2*, *Bmp6*, and *Smad7* mRNA levels compared to those of the VAD group (*p* < 0.001, 0.035, 0.0003, and 0.002, respectively; Fig. 1), whereas only *Bmp6* mRNA levels were significantly increased in the VAFeD group compared to those in the FeD group (*p* = 0.024).

### The effect of vitamin A deficiency and atRA administration on the *Fpn1* and *Dmt1* intestinal mRNA levels

**Fig. 2:**
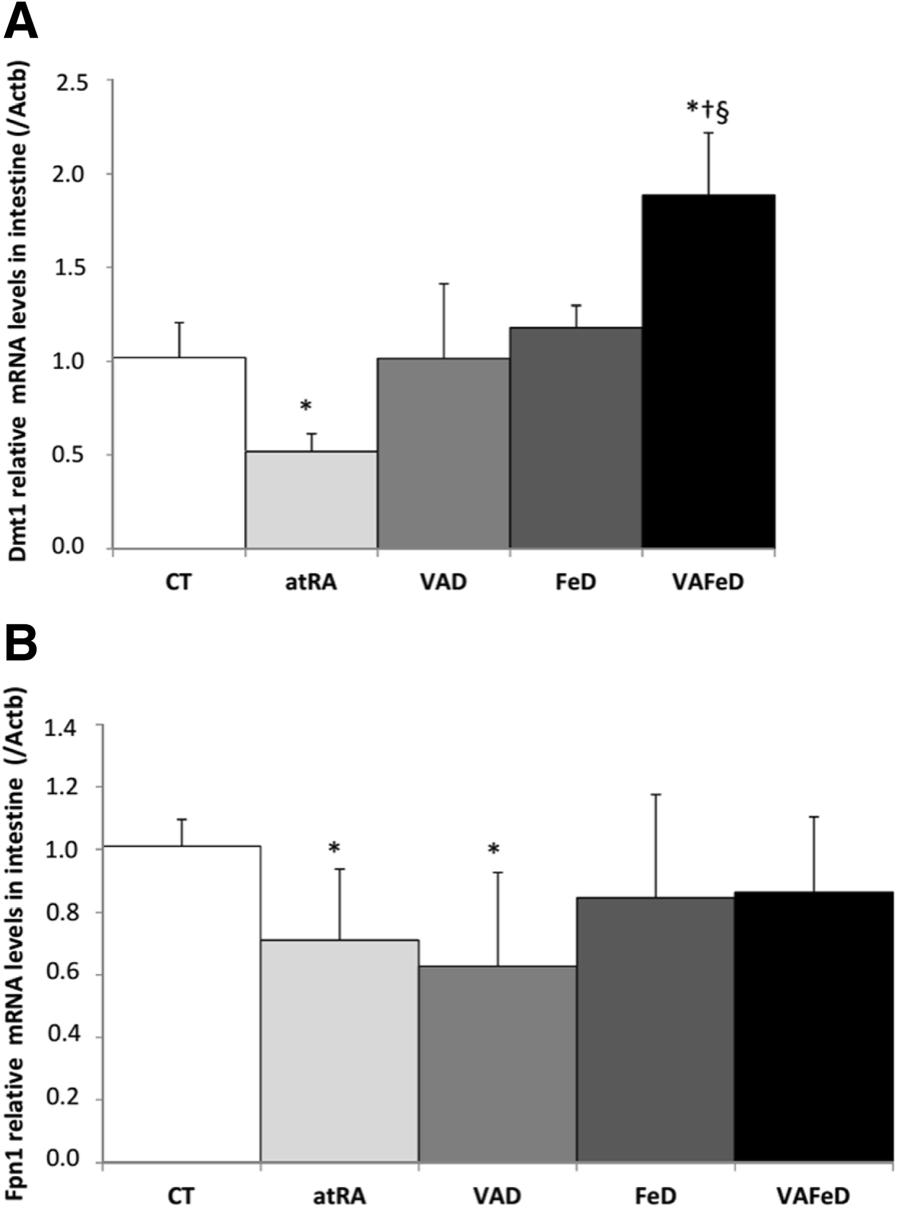
Relative transcript levels of divalent metal transporter 1 (*Dmt1*) (**a**) and ferroportin 1 (*Fpn1*) (**b**) in the small intestines normalized to β-actin (Actb) values of rats treated with diets containing different sources and amounts of vitamin A and iron for 59 days. Mean ± standard deviation, *p < 0.05 versus the CT group, †*p* < 0.05 versus the VAD group, §*p* < 0.05 versus the FeD group

Vitamin A deficiency promoted a decrease in *Fpn1* mRNA levels compared to the control group (1.6-fold, *p* = 0.043; Fig. 2). This response was not reversed when retinyl ester was replaced by all-trans retinoic acid in the diet; instead, the atRA group also showed down-regulation of *Fpn1* mRNA levels in the intestine relative to the control group (*p* = 0.020). The VAFeD group showed higher *Dmt1* mRNA levels than the control group (*p* = 0.001) and the FeD group (*p* = 0.002). No difference was observed on the *Dmt1* mRNA levels in vitamin A- or iron-deficient rats (VAD and FeD groups); however, the association of both deficiencies (VAFeD group) up-regulated *Dmt1* mRNA levels relative to the control (1.8-fold, *p* = 0.001), VAD (1.8-fold, *p* = 0.002), and FeD groups (1.5-fold, *p* = 0.002). The administration of all-trans retinoic acid (atRA) promoted the down-regulation of intestinal *Dmt1* mRNA levels compared to the control group (1.9-fold, *p* = 0.014; Fig. 2).

### The effect of vitamin A deficiency and atRA on the *Rarα* and *Rxrβ* mRNA levels in the liver

**Fig. 3:**
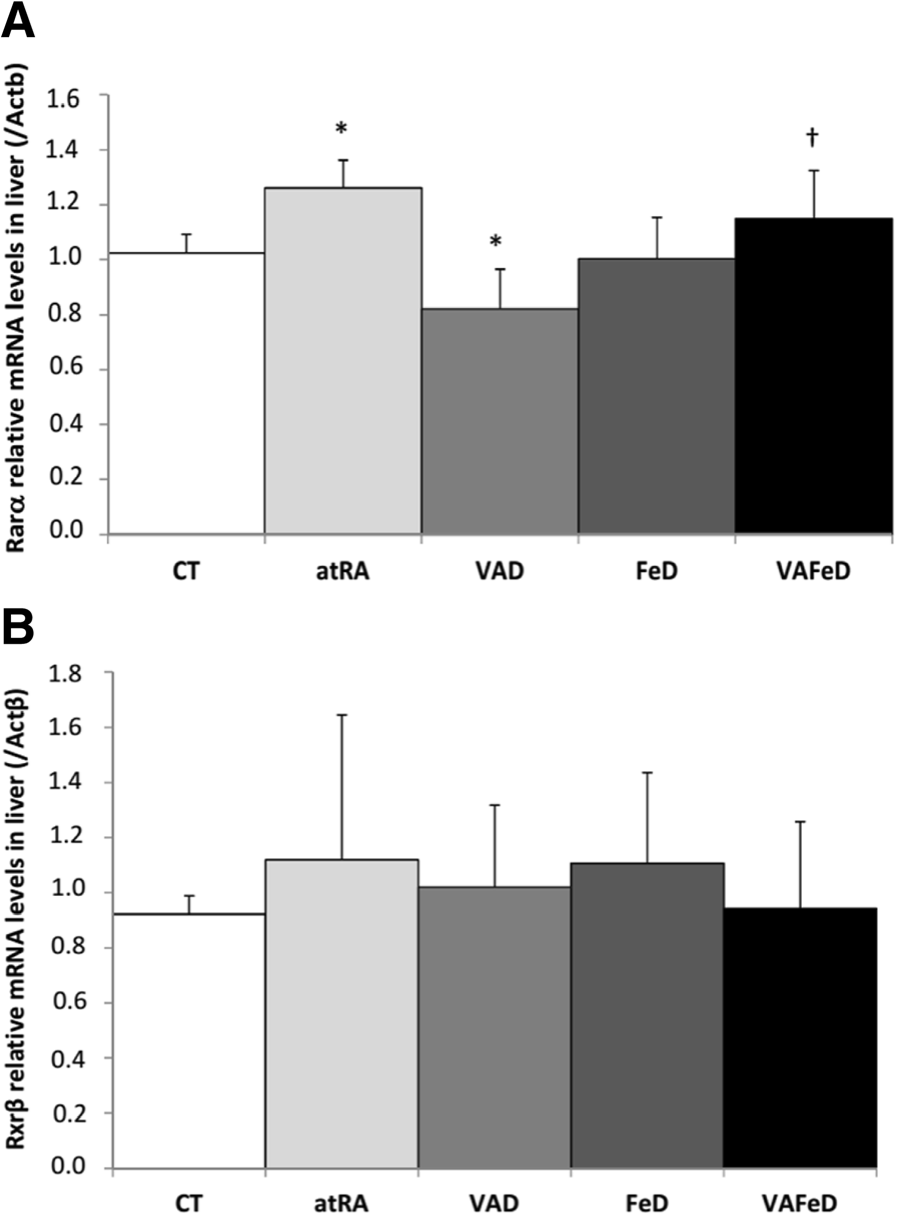
Relative transcript levels of retinoic acid receptor alpha (*Rarα*) (**a**) and retinoid X receptor beta (*Rxrβ*) (**b**) in the livers normalized to β-actin (Actb) values of rats treated with diets containing different sources and amounts of vitamin A and iron for 59 days. Mean ± standard deviation, *p < 0.05 versus the CT group, †*p* < 0.05 versus the VAD group, §*p* < 0.05 versus the FeD group

Vitamin A deficiency reduced the levels of hepatic *Rarα* mRNA (1.2-fold, *p* = 0.001), while the atRA diet up-regulated hepatic *Rarα* mRNA, in relation to the control rats. The association between vitamin A and iron deficiency (VAFeD) leads to higher hepatic *Rarα* mRNA levels compared with vitamin A deficiency (VAD), whereas no difference was observed in *Rarα* mRNA levels between the VAFeD and Fe groups. No differences were observed in hepatic *Rxrβ* mRNA levels among the groups (Fig. 3).

## Discussion

To explore how vitamin A deficiency alters hepcidin expression and consequently affects iron metabolism, the present study evaluated the expression of genes involved in the HJV-BMP6-SMAD signaling pathway in male Wistar rats treated with diets deficient in vitamin A (VAD) or in iron (FeD) or with diet deficient in both vitamin A and iron (VAFeD). Understanding the mechanisms that control hepcidin gene expression (*Hamp*) is an important goal in the treatment of anemia from chronic diseases.

The iron-deficient group (FeD) showed lower iron concentrations in the liver, spleen, and intestine and lower serum iron status compared to those of the control group (Additional file 1: Figure S1 and Additional file 2: Table S1). The tissue and systemic iron deficiency of FeD rats may have led to the lower levels of hepatic *Bmp6* mRNA and consequent strong down-regulation of *Hamp* mRNA levels compared to those of the control group. These results are consistent with a previous study (Pagani et al. 2011) that found that iron deficiency inhibits hepcidin due to the inhibition of the BMP-SMAD pathway as a consequence of reductions in *Bmp6* mRNA. Corradini et al. (2011) suggested that liver iron concentration was the only factor associated with hepatic *Bmp6* mRNA levels and that it is independent of transferrin saturation and serum iron and hemoglobin concentration, which suggests that hepatic *Bmp6* induction is mediated by liver iron concentration but not by transferrin saturation. Despite hemojuvelin (HJV) acting as a BMP co-receptor in the BMP-signaling pathway that modulates hepatic hepcidin expression (Evstatiev and Gasche 2012), the iron deficiency (FeD) did not alter hepatic *Hfe2* mRNA levels compared with those of the control. Krijt et al (2012) observed no changes in membrane HJV protein levels in rat livers subjected to iron overload and deficiency. The authors suggested that substantial changes in *Hamp* mRNA can occur without changes in membrane hemojuvelin content and that this protein is not a limiting step in the control of *Hamp* gene expression.

Although the vitamin A-deficient rats (VAD) have presented systemic iron deficiency (lower serum iron concentration and transferrin saturation) like the iron-deficient groups (FeD and VAFeD), they showed iron retention in the spleen instead of a decrease as would be expected (Additional file 1: Figure S1 and Additional file 2: Table S1). Further, the vitamin A deficiency up-regulated hepatic *Bmp6* and *Hfe* mRNA levels in relation to those of the control group and down-regulated hepatic *Hamp* mRNA levels, despite similar iron levels in the liver, compared to those of the control group (Fig. 1). This contradictory response observed in the VAD group may be associated to an inadequate erythropoiesis promoted by vitamin A deficiency, due to the low availability of iron to expand the erythroid tissue (Evans 2005; Frazer et al. 2012). The inadequate erythropoiesis was characterized in a set of other experiments conducted in these rats (Cunha et al. 2014), where the authors demonstrated that vitamin A deficiency down-regulated renal *Epo* mRNA and up-regulated *Hmox1* in the spleen, suggesting an increased phagocytosis of malformed or undifferentiated erythrocytes and consequent iron accumulation in the spleen, in the heme form. Frazer et al. (2012) observed that a chronic stimulated erythropoiesis led to an increase in liver iron and *Bmp6* mRNA levels and an unexpected decrease in hepatic *Hamp* mRNA levels. A similar profile was also observed in a model of hemolytic anemia, low serum iron status, and high tissue iron concentration, where a reduction in hepcidin expression occurs despite an increase in *Bmp6* expression (Frazer et al. 2012).

Despite all-trans retinoic acid (atRA) being considered the most active form of retinoids, rats treated with 12 mg/kg of atRA to replace retinyl ester presented decreased spleen, gut, and serum iron levels and low transferrin concentrations, in spite of their high liver iron store in relation to that of the control group (Additional file 1: Figure S1 and Additional file 2: Table S1). The low intestinal iron level may lead to the down-regulation of *Fpn1* mRNA levels in the atRA, since during cellular iron deprivation the iron regulatory protein (IRP1 or IRP2)/iron responsive element (IRE) system (IRP/IRE) represses ferroportin translation to reduce iron exportation and preserve cellular iron (Jiang et al. 2012; Galy et al. 2013; Sangokoya et al. 2013). Moreover, the atRA diet did not restore the levels of hepatic *Hamp* mRNA in the atRA group (Fig. 1) compared to those in the control group, and the expression of hepatic *Bmp6* and *Hfe2* genes that are involved in the activation of hepatic hepcidin expression through the BMP-SMAD-HJV pathway was similar to that obtained for the control group. The low systemic iron status of the atRA group in the present study may be responsible for the lower *Hamp* mRNA levels in the liver. Altogether, these contradictory results suggest that the single atRA dose used (12 mg/kg diet) must be suboptimal, either being too high or too low for the physiological needs of rats. Deviations in both directions are likely to induce an abnormal response (Martini et al. 1995).

Different proteins have been identified as negative regulators of hepcidin transcription, including the small mothers against decapentaplegic protein 7 (SMAD 7), which mediate a negative feedback response through the BMP pathway (Mleczko-Sanecka et al. 2010). SMAD 7 and *Hamp* are coregulated in the liver of iron-loaded mice, which suggests that a negative feedback loop is initiated by activating signals (Kautz et al. 2008). Consistent with the coregulation that has been proposed in the literature, the lower levels of hepcidin mRNA that were found in the FeD group were accompanied by down-regulation of hepatic *Smad7* mRNA levels, which corroborates the idea that systemic and tissue iron deficiency lead to low hepcidin concentrations and consequently decrease *Smad7* expression, the inhibitor of the BMP pathway. The down-regulation of hepatic *Smad7* mRNA levels in the VAD group may reflect the inhibition of hepcidin by the systemic iron deficiency, as the levels of *Smad7* and *Hamp* are coregulated in the liver. The association of both deficiencies (VAFeD group) reduced the iron concentrations in the liver, intestine, and spleen (Additional file 1: Figure S1), which may explain the down-regulation of *Hamp*, *Hfe2*, *Bmp6*, and *Smad7* hepatic mRNA levels compared to that of the VAD group (Fig. 1). These results suggest that iron plays a central role in the modulation of the expression of these genes.

Contrary to the present study, in our previous study (Arruda et al. 2009), an up-regulation of *Hamp* expression in VAD rats was observed. However, these apparent contradictory results may be explained by differences in rats’ treatment. In the previous study, during the acclimatization period (8 days), the rats were maintained in a commercial rodent diet (Labina Purina/Brazil) with a concentration of vitamin A and iron six- and fivefold higher than the AIN-93G standardized diet, respectively. Considering that vitamin A is fat-soluble, these rats might have higher vitamin A content in the liver at the beginning of the experiment, which may have led to a late establishment of vitamin A deficiency and consequent difference in *Hamp* response, between the studies.

Besides the post-transcriptional regulation of intestinal ferroportin by the iron regulatory protein/iron responsive element (IRE) system in response to cellular iron content, duodenal iron absorption and exportation is also dramatically altered in response to changes in erythropoietic signal and hematological parameters. In the present study, the decrease in intestinal ferroportin mRNA levels in the vitamin A-deficient group may be associated with the decrease in kidney erythropoietin mRNA levels (Cunha et al. 2014) and decreased serum iron status once iron absorption and exportation is altered in response to changes in erythropoietic signal and hematological parameters. Srai et al (2010) showed that a nephrectomy rat model of chronic renal failure had decreased iron transport across the duodenal epithelium compared with sham-operated controls, and the treatment of these rats with Epo significantly increased iron absorption. Their findings suggest that Epo acts directly on intestinal enterocytes to regulate dietary absorption. Similar results were observed in Caco-2 cells treated with Epo, where higher protein and mRNA levels of intestinal DMT-1 and FPN led to increased iron uptake and efflux compared to those of the control cells.

Considering that a transgenic mouse, in which retinoid signaling is impaired specifically in hepatocytes, showed increased expression of hemojuvelin and hepcidin (Tsuchiya et al. 2009), we investigated if modulation of *Hamp* mRNA levels by vitamin A involves retinoic acid receptor (*Rarα*) or retinoid X receptor beta (*Rxrβ*). Although the atRA diet promoted up-regulation and the VAD diet promoted down-regulation of retinoic acid receptor (*Rarα*) mRNA levels in the liver, in both treatments, *Hamp* mRNA levels were reduced (Fig. 3). These results suggest that vitamin A deficiency influences hepcidin expression in an indirect way that does not involve the hepatic *Rarα* receptor.

## Conclusions

In conclusion, this study demonstrates that the HJV-BMP6-SMAD signaling pathway that normally activates the expression of hepcidin in iron deficiency is impaired by vitamin A deficiency despite increased expression of liver *Bmp6* and *Hfe2* mRNA levels and decreased expression of Smad7 mRNA. The impairment of HJV-BMP6-SMAD signaling pathway may be associated to the systemic iron deficiency and spleen iron retention promoted by vitamin A deficiency.

## Additional files

Additional file 1: Figure S1. Iron concentration in the liver (A), small intestine (B), and spleen (C) of rats treated with diets containing different sources and amounts of vitamin A and iron for 59 days. Mean ± standard deviation, * p < 0.05 versus the CT group, † p < 0.05 versus the VAD group, § p < 0.05 versus the FeD group. (DOCX 126 kb) Additional file 2: Table S1. Hematological parameters of rats treated with diets containing different sources and amounts of vitamin A and iron, for 59 days. (DOCX 30.5 kb)
