# Supplementary material for: Vitamin A deficiency modulates iron metabolism independent of hemojuvelin (Hfe2) and bone morphogenetic protein 6 (Bmp6) transcript levels
Source: Genes Nutr. 2016 Mar 17;11:1. doi: 10.1186/s12263-016-0519-4 (PMC4968453; doi:10.1186/s12263-016-0519-4)

**Vitamin A deficiency modulates iron metabolism independent of hemojuvelin (*Hfe2*) and bone morphogenetic protein-6 (*Bmp6*) transcript levels.**

Gene & Nutrition

Juliana Frossard Ribeiro Mendes^1*^, Egle Machado de Almeida Siqueira^2^, João Gabriel Marques de Brito e Silva**^3^**, Sandra Fernandes Arruda^4^

^1^Postgraduate Program in Human Nutrition, Faculty of Health Sciences, University of Brasília. [jufrossard@gmail.com](mailto:jufrossard@gmail.com)

^2^Cell Biology Department of Biological Sciences Institute, University of Brasília. [eglemasi@gmail.com](mailto:eglemasi@gmail.com)

^3^Nutrition Department of Health Sciences Faculty, University of Brasília. [jgmarques27@gmail.com](mailto:jgmarques27@gmail.com)

^4^Postgraduate Program in Human Nutrition, Faculty of Health Sciences, University of Brasília. [sandrafarruda@gmail.com](mailto:sandrafarruda@gmail.com)

^1, 2, 3, 4^ Campus Universitário Darcy Ribeiro, Universidade de Brasília, Brasília, DF, Brazil. POBox 70910- 900.

*Corresponding author:

Juliana Frossard Ribeiro Mendes. E-mail [jufrossard@gmail.com](mailto:jufrossard@gmail.com); Phone +55 61 31073099 or 3107 3100; Fax + 55 61 3273 3676. Universidade de Brasília, Campus Universitário Darcy Ribeiro, Instituto de Ciências Biológicas, Departamento de Biologia Celular, Laboratório de Bioquímica da Nutrição, Bloco J, 1° Andar. Asa Norte, Distrito Federal, Brasil. CEP: 70910.900


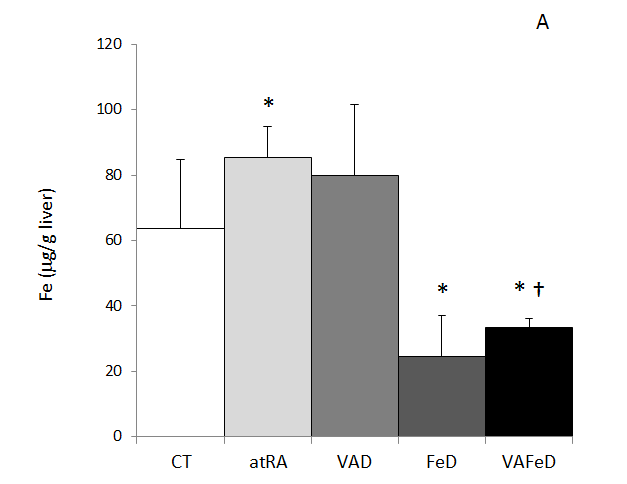

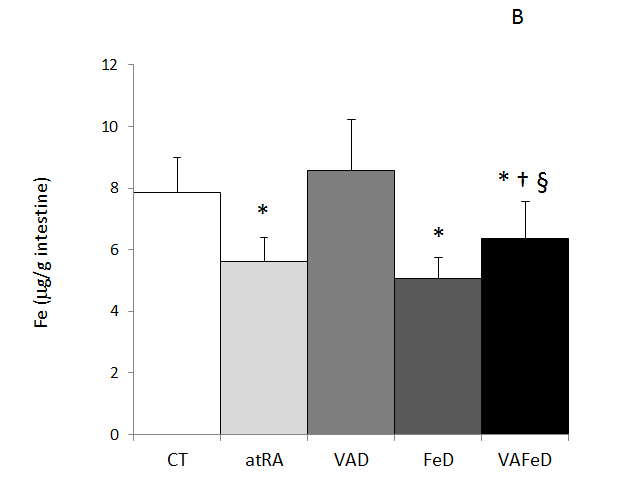

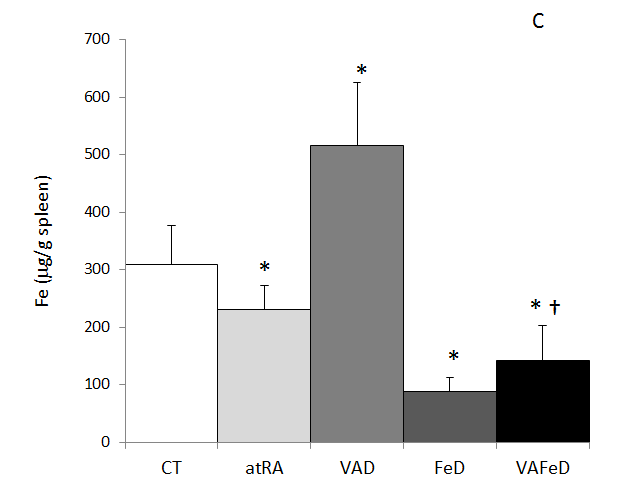

Supplement: Additional file 1: Figure S1. — Iron concentration in the liver (A), small intestine (B), and spleen (C) of rats treated with diets containing different sources and amounts of vitamin A and iron for 59 days. Mean ± standard deviation, * p < 0.05 versus the CT group, † p < 0.05 versus the VAD group, § p < 0.05 versus the FeD group. (DOCX 126 kb) [file 12263_2016_519_MOESM1_ESM.docx]
