# Supplementary material for: Vitamin A deficiency modulates iron metabolism independent of hemojuvelin (Hfe2) and bone morphogenetic protein 6 (Bmp6) transcript levels
Source: Genes Nutr. 2016 Mar 17;11:1. doi: 10.1186/s12263-016-0519-4 (PMC4968453; doi:10.1186/s12263-016-0519-4)
Supplement: Additional file 2: Table S1. — Hematological parameters of rats treated with diets containing different sources and amounts of vitamin A and iron, for 59 days. (DOCX 30.5 kb) [file 12263_2016_519_MOESM2_ESM.docx]

**Vitamin A deficiency modulates iron metabolism independent of hemojuvelin (*Hfe2*) and bone morphogenetic protein-6 (*Bmp6*) transcript levels.**

Gene & Nutrition

Juliana Frossard Ribeiro Mendes^1*^, Egle Machado de Almeida Siqueira^2^, João Gabriel Marques de Brito e Silva**^3^**, Sandra Fernandes Arruda^4^

^1^Postgraduate Program in Human Nutrition, Faculty of Health Sciences, University of Brasília. [jufrossard@gmail.com](mailto:jufrossard@gmail.com)

^2^Cell Biology Department of Biological Sciences Institute, University of Brasília. [eglemasi@gmail.com](mailto:eglemasi@gmail.com)

^3^Nutrition Department of Health Sciences Faculty, University of Brasília. [jgmarques27@gmail.com](mailto:jgmarques27@gmail.com)

^4^Postgraduate Program in Human Nutrition, Faculty of Health Sciences, University of Brasília. [sandrafarruda@gmail.com](mailto:sandrafarruda@gmail.com)

^1, 2, 3, 4^ Campus Universitário Darcy Ribeiro, Universidade de Brasília, Brasília, DF, Brazil. POBox 70910- 900.

*Corresponding author:

Juliana Frossard Ribeiro Mendes. E-mail [jufrossard@gmail.com](mailto:jufrossard@gmail.com); Phone +55 61 31073099 or 3107 3100; Fax + 55 61 3273 3676. Universidade de Brasília, Campus Universitário Darcy Ribeiro, Instituto de Ciências Biológicas, Departamento de Biologia Celular, Laboratório de Bioquímica da Nutrição, Bloco J, 1° Andar. Asa Norte, Distrito Federal, Brasil. CEP: 70910.900.

**Table S1** Hematological parameters of rats treated with diets containing different sources and amounts of vitamin A and iron, for 59 days

| **CT** | **Serum iron**  (μg/dL) | **UIBC**  (μg/dL) | **TIBIC**  (μg/dL) | **TS**  (%) |
| --- | --- | --- | --- | --- |
|  | 161.41 ± 42.01 | 291.03 ± 43.68 | 452,45 ± 47.73 | 35.60 ± 8.05 |
| **atRA** | 100.11 ± 28.26 * | 307.31 ± 74.16 | 406.46 ± 68.12 | 25.39 ± 9.19 |
| **VAD** | 93.55 ± 10.72 * | 373.74 ± 49.56 * | 484.84 ± 67.09 | 22.65 ± 6.46* |
| **FeD** | 78.53 ± 22.65 * | 409.01 ± 84.36 * | 487.54 ± 68.41 | 16.75 ± 6.77* |
| **VAFeD** | 109.53 ± 28.53*§ | 380.45 ± 105.77 | 489.98 ± 88.28 | 19.85 ± 4.51* |

UIBC, unsaturable iron-binding capacity; TIBC, total iron binding capacity; TS, transferrin saturation. Mean ± standard deviation. * p < 0.05 versus CT group; † p < 0.05 versus VAD group; § p < 0.05 versus FeD group
